# A restriction enzyme reduced representation sequencing approach for low-cost, high-throughput metagenome profiling

**DOI:** 10.1371/journal.pone.0219882

**Published:** 2020-04-03

**Authors:** Melanie K. Hess, Suzanne J. Rowe, Tracey C. Van Stijn, Hannah M. Henry, Sharon M. Hickey, Rudiger Brauning, Alan F. McCulloch, Andrew S. Hess, Michelle R. Kirk, Sandeep Kumar, Cesar Pinares-Patiño, Sandra Kittelmann, Graham R. Wood, Peter H. Janssen, John C. McEwan

**Affiliations:** 1 AgResearch Limited, Invermay Agricultural Centre, Mosgiel, New Zealand; 2 AgResearch Limited, Ruakura Agricultural Centre, Hamilton, New Zealand; 3 AgResearch Limited, Grasslands Research Centre, Palmerston North, New Zealand; University of Illinois, UNITED STATES

## Abstract

Microbial community profiles have been associated with a variety of traits, including methane emissions in livestock. These profiles can be difficult and expensive to obtain for thousands of samples (e.g. for accurate association of microbial profiles with traits), therefore the objective of this work was to develop a low-cost, high-throughput approach to capture the diversity of the rumen microbiome. Restriction enzyme reduced representation sequencing (RE-RRS) using *ApeK*I or *Pst*I, and two bioinformatic pipelines (reference-based and reference-free) were compared to bacterial 16S rRNA gene sequencing using repeated samples collected two weeks apart from 118 sheep that were phenotypically extreme (60 high and 58 low) for methane emitted per kg dry matter intake (n = 236). DNA was extracted from freeze-dried rumen samples using a phenol chloroform and bead-beating protocol prior to RE-RRS. The resulting sequences were used to investigate the repeatability of the rumen microbial community profiles, the effect of laboratory and analytical method, and the relationship with methane production. The results suggested that the best method was *Pst*I RE-RRS analyzed with the reference-free approach, which accounted for 53.3±5.9% of reads, and had repeatabilities of 0.49±0.07 and 0.50±0.07 for the first two principal components (PC1 and PC2), phenotypic correlations with methane yield of 0.43±0.06 and 0.46±0.06 for PC1 and PC2, and explained 41±8% of the variation in methane yield. These results were significantly better than for bacterial 16S rRNA gene sequencing of the same samples (p<0.05) except for the correlation between PC2 and methane yield. A Sensitivity study suggested approximately 2000 samples could be sequenced in a single lane on an Illumina HiSeq 2500, meaning the current work using 118 samples/lane and future proposed 384 samples/lane are well within that threshold. With minor adaptations, our approach could be used to obtain microbial profiles from other metagenomic samples.

## Introduction

Metagenomics is the study of genetic material recovered directly from environmental samples and captures the myriad of organisms present in that environment. Samples of soil and water are obvious examples of environmental samples, but the gastro-intestinal tract can also be considered an environment due to the presence of microbes that interact with the feed and host, e.g. during digestion. Metagenomic studies have gained popularity in recent years, primarily in human health, e.g. Irritable Bowel Disease [1] and Coeliac Disease [2].

In an agricultural setting, rumen microbial community (RMC) profiles have been associated with environmentally and economically important traits, such as methane emissions [3, 4] and feed efficiency [5, 6]. The bacteria, protozoa and fungi of the RMC breaks down ingested feed to produce volatile or short chain fatty acids, which are a source of energy for the host. Hydrogen, formate and methyl compounds produced by this process are growth substrates for methanogenic archaea (methanogens) in the RMC, and are metabolized by them into methane. Methane production is not truly dependent on the abundance of methanogens present, but instead on the amounts of their substrates available to them [7]. Different “ruminotypes”, generalized classifications of individual RMC types, can be found in sheep with high and low methane production, with at least two ruminotypes present in low-methane sheep fed lucerne pellets [3]. These are postulated to ferment the feed in different ways, producing different amounts of the substrates for methanogens [8], indicating that the RMC is related to the amount of methane emitted.

RMC profiles from rumen samples are moderately heritable [9], suggesting that selection of parents based on RMC profiles is likely to result in changes in offspring microbiomes. Given that traits such as methane emissions and feed efficiency are difficult and expensive to measure, selection on RMC profiles may facilitate a reduction in the environmental impact of livestock, provided costs are low enough and the method is high-throughput.

Historically, there have been two approaches used for sequencing metagenome samples: targeted sequencing and metagenome shotgun sequencing. Targeted sequencing amplifies specified phylogenetically informative genes from a sample, such as the 16S rRNA gene (16S) of microbes, which typically distinguishes taxonomic groups well due to large, comprehensive databases of 16S rRNA sequences that include both culturable and uncultured organisms [10, 11]. This approach usually relies on having long sequence reads [12], only captures phylogenetic variation at one gene, and is subject to PCR primer bias due to mismatches in the flanking regions where the primers bind [13]. Metagenome shotgun sequencing can capture any part of the microbial, host or feed genome; but a reference database of genome assemblies with known taxonomies, e.g. the Hungate1000 Collection [14], is needed to obtain taxonomic information on metagenome shotgun sequences to bin sequences into informative groups. Whole genome assemblies are historically difficult to obtain on uncultured microbes, so these are largely missing from reference databases [15]. Hundreds of millions of reads are generated per sample for metagenome shotgun sequencing, making it an expensive and time-consuming method that additionally requires significant computation resources.

Restriction Enzyme-Reduced Representation Sequencing (RE-RRS, also known as Genotyping-by-Sequencing or GBS) is a next-generation sequencing technique that reduces genome complexity by digestion of genomic DNA by restriction enzymes, followed by the sequencing of fragments within a given size range [16]. RE-RRS is used to obtain genotypes for parentage identification or genomic selection (i.e. to identify the individuals with the most favorable genotypes associated with phenotypes of interest) in a variety of species across livestock, plants and aquaculture [15–18], as well as population diversity studies, e.g. for conservation [17].

RE-RRS holds promise as a technique for rapid, high-throughput and cost-effective sequencing of metagenome samples at a fraction of the cost of metagenome shotgun sequencing. Underlying the RE-RRS method is the assumption that sequencing only a specific fraction, typically 0.5–1% of any microbial genome as defined by restriction site and fragment size, captures the majority of information on composition and diversity of the microbial community at a fraction of the sequencing cost. Unlike 16S rRNA sequencing, RE-RRS is not limited to capturing organisms with a particular gene and is therefore able to capture a wider variety of organisms, e.g. host, viruses, fungi, that may contribute to the trait of interest e.g. methane yield. This study used sheep rumen samples to show the potential of RE-RRS as a low-cost, high-throughput approach for obtaining metagenome profiles on thousands of samples, and describes pipelines for obtaining profiles both with and without a reference database.

## Materials and methods

Samples used in this study were collected as part of the study published by Kittelmann et al. [3]. The use of animals, including welfare, feeding, experimental procedures, and the collection of rumen samples used for this study, was approved by the AgResearch Grasslands Animal Ethics Committee (Application number 11975), and complied with the institutional Codes of Ethical Conduct for the Use of Animals in Research, Testing and Teaching, as prescribed in the New Zealand Animal Welfare Act of 1999 and its amendments.

### Rumen sampling and associated methane yields

The sheep rumen samples and methane yield data used for this study were those for which RMC structure was analyzed using 16S rRNA gene sequencing in Kittelmann et al. [3] and part of a larger experiment described in Pinares-Patiño et al. (18). Briefly, respiration chambers were used to measure methane yield (g CH_4_/ kg DMI) on 340 sheep at two independent measuring rounds two weeks apart, each over two days in 4 separate cohorts of animals. The rumen sample was collected via stomach tubing at the end of each measuring round and immediately stored at −20°C. Two rumen samples from a subsample of 118 sheep (n = 236), representing the ~17% highest and lowest emitters (60 high-methane sheep and 58 low-methane sheep based on methane yield phenotype), were previously freeze dried, homogenized and stored at −85°C.

### Reanalysis of bacterial 16S rRNA gene sequence data

Subsamples of the extracted rumen contents were previously used for analysis of RMC by pyrosequencing amplified bacterial 16S rRNA genes, and these sequences are available in the EMBL database under the study accession number ERP003779 [3]. The sequences were reanalyzed using the QIIME pipeline v1.5.0 [19]. The reads were first checked for quality using FastQC [20] and assigned to their respective biological samples using nucleotide barcodes. Only sequences >400 bp with a quality score over 27 (sliding window 50 bases) along the whole sequence were included for analysis. The pyrosequencing dataset was denoised using Acacia [21]. Denoised sequences were grouped into OTUs (Operational Taxonomic Units) and from each OTU, one representative sequence was selected and designated the repset sequence for that OTU. These repset sequences were compared in QIIME against reference sequences in an improved bacterial taxonomic framework [11], using BLASTN and a default e-value cut-off of 0.001. In this step, each repset sequence was assigned to its closest relative in the taxonomy framework. In this way, the sequences in each OTU were assigned to a bacterial taxon, with multiple OTUs with highly similar repset sequences being assigned to the same taxon. The bacterial genus level was chosen as the taxonomic rank to summarize the repset data. These same freeze-dried and homogenized samples were used in our study to evaluate the potential of using RE-RRS for RMC profiling, as described below.

### DNA extraction and restriction enzyme-reduced representation sequencing

DNA was extracted from the 236 rumen samples using a combined bead-beating, phenol and column purification protocol, as described in Text S1 of Kittelmann et al. [3], to provide high quality nucleic acids for RE-RRS. *Ape*KI and *Pst*I restriction enzymes were used separately to test whether RE-RRS is a suitable approach for rumen metagenome profiling. These two enzymes were selected because an in silico digestion and size filtering (65–195 bp) of rumen microbial genome assemblies from the Hungate1000 Collection [14] showed that RE-RRS using either *Ape*KI (G|CWGC) or *Pst*I (CTGCA|G) captured microbial sequence from all species present in the collection with an average of 8.6% and 0.3% of each genome, respectively [22]. These two restriction enzymes represent two different approaches to capturing the RMC profile at the same sequencing cost: more of the genome captured at lower depth (*Ape*KI) and less of the genome captured at higher depth (*Pst*I).

After digestion of DNA by either *Ape*KI or *Pst*I, barcodes were ligated to link sequences to samples, as described by Elshire et al. [16], and samples were grouped into two libraries, one library for each restriction enzyme used. Pooled libraries were purified through a QIAquick 96 PCR Purification Kit (Qiagen, Hilden, Germany), the elute was then PCR amplified using the PCR primers and conditions outlined in Elshire et al. [16]. A Pippin Prep (SAGE Science, Massachusetts, USA) was used with “Narrow” settings (193–318 bp, corresponding to 65–195 bp inserts) for size selection of amplified sequences. Each library was checked on a High Sensitivity DNA chip (Agilent Bioanalyzer) then run on two lanes on the same flow cell on an Illumina HiSeq2500 machine, generating 101 bp single end reads using version 4 chemistry. One plate of 94 samples for *Pst*I were re-run (in a single lane) because barcodes did not ligate in the initial run. FastQ files were deposited in the NCBI SRA under BioProject ID PRJNA607369. S1 Table links sample accession numbers for 16S rRNA gene sequences and RE-RRS sequences along with sequencing statistics.

### Bioinformatic pipeline

Sequenced reads were demultiplexed using GBSX [23], and trimmed using trim_galore [24] for single reads with a minimum length of 40 base pairs. Samples with fewer than 100,000 reads across both lanes of sequencing for a single restriction enzyme were removed from all further analyses, consisting of one sample for *Ape*KI and two samples for *Pst*I. The trimmed sequences from the remaining samples were run through both the reference-based and reference-free pipelines, described below. The proportion of trimmed sequences that mapped to the host genome was evaluated using BWA mem [25] with the *Ovis aries* 3.1 genome assembly (https://www.ncbi.nlm.nih.gov/assembly/GCF_000298735.1/), by considering those that returned a flag of 0 or 16, referring to those that uniquely mapped in the forward and reverse orientation, respectively.

### Reference-based approach

The reference-based (RB) approach used nucleotide BLAST (BLASTN) in BLAST v2.2.28+ [26] with default parameters apart from task set to blastn and word size of 16, to compare sequenced reads against the 410 rumen microbial genome assemblies from the Hungate1000 Collection [14]. A variety of nucleotide BLAST parameters were evaluated, and these parameters were found to be optimal for aligning query sequences to the Hungate1000 Collection (S1 File). Reads were assigned to a taxonomic node using the algorithm from MEGAN [27] implemented in R with default parameters: a minimum bitscore of 50 and considering only hits within 10% of the maximum bitscore for a query read. This approach was found to assign reads at the genus level with high accuracy (>95%; S1 File). The RMC profile was defined as the number of sequences assigned to each of the ~60 genera represented in the Hungate1000 Collection and associated analyses will be denoted by *Ape*KI_RB and *Pst*I_RB for analyses of *Ape*KI and *Pst*I profiles, respectively.

### Reference-free approach

The reference-free approach involved collating a set of “tags”, i.e. non-redundant 65 bp-long DNA sequences (evaluated across all samples) commencing at the initial cut site, using an in-house Unix script. The RMC profile for each sample was generated by counting the abundance of each tag from the sequenced reads; these profiles were collated into a count matrix with samples as rows and tags as columns, obtained using an in-house Unix script. Tags were required to be present in 25% of samples and a comparison of performance for other tag lengths (16, 32 or 65 bp) and a variety of prevalence thresholds (10%, 25%, 50%, 100%) can be found in S2 File. Requiring 65 bp tags to be present in at least 25% of samples gave high estimates with low standard errors for repeatability, correlation with methane yield and microbiability, so these parameters were selected for subsequent analyses. Reference-free analyses will be denoted as *Ape*KI_RF and *Pst*I_RF for profiles from the *Ape*KI and *Pst*I restriction enzymes, respectively.

The reference-free approach will capture sequences from a wider taxonomic range than just bacteria and archaea. The 65 bp tags present in at least 25% of samples were compared against the GenBank database [28] using BLASTN with the task set to blastn and a word size of 11 to evaluate the taxonomic range that was captured by RE-RRS. Taxonomy was assigned to tags based on the algorithm described for the reference-based approach, with a minimum bitscore of 50 and considering only hits with the maximum bitscore for that tag. These parameters differed from those used for the reference-based approach because we were more interested in assigning taxonomy for as many tags as possible at higher taxonomic levels (e.g. Kingdom to Order levels), rather than highly accurate assignment at the Genus level (S1 File). Runtime was also less important because it was a BLAST of 233,587 (*Ape*KI) or 502,900 (*Pst*I) 65 bp sequences rather than hundreds of millions of 40–92 bp reads.

### Comparison of methods for obtaining RMC profiles

#### Parameter estimation from principal component analysis

A principal component analysis was used to reduce the dimensionality of the dataset and facilitate comparisons between the different methods for generating RMC profiles. The count matrix was transformed into a matrix of log_10_ proportions by dividing each count by the rowsum (representing the number of reads that had been assigned for that sample) and taking the log_10_ of this proportion. The principal component analysis was performed of the matrix of log_10_ proportions using the prcomp command in R with scale = TRUE, which gives each genus (RB) or tag (RF) an equal weight.

Repeatability of the first two principal components (PC1 and PC2), and their phenotypic correlation with scaled methane yield were estimated in ASReml 4.1 [29]. Repeatability and the proportion of the variance in the principal component explained by the cohort effect were estimated using a univariate mixed linear model, and the correlation between each principal component and scaled methane yield was estimated with a bivariate mixed linear model. Scaled methane yield was obtained by dividing methane yield by the contemporary group mean and multiplying by the overall mean, where contemporary group included recording year, lot (mob of 96 animals), group (sub-mob of up to 24 animals within a lot, measured contemporaneously) and round (measurement time, 14 days apart) and the overall mean was 16.0 g CH_4_/kg dry matter intake, as described in Pinares-Patiño et al. [18]. In both univariate and bivariate models, cohort (lot and round) was fitted as a fixed class effect, and a random permanent environmental effect linked duplicate samples from the same animal.

### Microbiability

The microbiability is the percent of the variance in a trait, in our case methane yield, that can be explained by a microbial profile [30]. Estimates of microbiability were obtained by fitting a univariate model in ASReml 4.1 [29] with scaled methane yield as the dependent variable, fitting the mean and two random effects: the RMC profile based on a microbial relationship matrix (MRM) and a random permanent environmental effect. The MRM was generated by normalizing each column of the log_10_ proportion matrix within cohort (mean = 0, sd = 1) and generating a correlation matrix using the cor function in R, generating an n×n MRM where n is the number of samples. The microbiability was estimated as the proportion of the phenotypic variance that was attributed to the MRM.

### Sensitivity to sequencing depth

Sequencing more samples per lane would lower the cost of RE-RRS profiling but would consequently reduce the sequencing depth. At low depths the profiling might not accurately capture the proportion of each microbe in the sample, particularly microbes that are in low abundance. Therefore, a sensitivity analysis was performed to evaluate the impact of reducing the sequencing depth in our approach. Reads were subsampled with probability 0.5, 0.25, 0.1, 0.05, 0.01, 0.005, 0.002 or 0.001 using the sample function in R; representing sequencing 2, 4, 10, 20, 100, 200, 500 or 1000 times the number of samples per lane, respectively. The set of sampled reads at a given simulated sequencing depth were then used to calculate compression efficiency [31]. Compression efficiency compares the size of a compressed file to its original size as (original–compressed)/original and is a measure of the non-redundant information present in the file. In our study, the original file contained the reads for a given sample without their identifiers. This file was compressed using gzip 1.3.12 [32], which uses the DEFLATE algorithm [33]. The value of compression efficiency was the mean across all samples for the simulated sequencing depth. Standard errors were the standard deviation across five replicates at that sequencing depth.

## Results and discussion

### Sequencing results

Sequence read quality was high for all lanes of DNA prepared for RE-RRS (S1 Fig). A greater average number of reads per sample was observed for samples digested with *Pst*I (Table 1), likely partially due to re-running of samples–only 94 samples were run in that lane rather than 118 (i.e. 236 samples across 2 lanes). Sequences from the *Ape*KI digest were slightly shorter than from the *Pst*I digest, but this difference was not significant based on a t-test with alpha = 0.05.

**Table 1:** Average number of reads per sample and average read length of RE-RRS reads.

Restrictionenzyme	Reads per sample (sd)	Read length (sd)^1^
*Ape*KI	2.4M (870k)	71 (17)
*Pst*I	2.7M (680k)	84 15)

1. Trimmed read length in base pairs after barcode removed.

The proportion of reads that mapped to the sheep genome was generally low, at 0.34 ± 0.85% for *Ape*KI and 0.94 ± 2.11% for *Pst*I; however, one outlier sample had the highest percent of reads mapping to the sheep genome for both restriction enzymes: 11.52% with *Ape*KI and 25.55% with *Pst*I. The low proportion of reads mapping to the host genome means that we can ignore the proportion of reads mapping to the host when calculating the performance of the reference-free and reference-based pipelines.

### Reference-based approach

Using the MEGAN algorithm on nucleotide BLAST results, 18.7 ± 3.3% and 23.4 ± 3.7% of reads were assigned at the genus level for *Ape*KI and *Pst*I, respectively. Comparing against a protein database is one method that could potentially improve the proportion of sequences assigned (hit rate) at the genus level. Hess et al. [22] found a small increase in hit rate at the genus level when BLASTX was used (BLASTN = 8.8%, BLASTX = 11.5%; averaged across high- and low- methane samples, both with default parameters), but a much longer runtime to perform the BLAST query and analyze the results of 1 million query sequences when using BLASTX (11 days) rather than BLASTN (15 minutes). They therefore determined that BLASTX was not desirable for a high-throughput pipeline.

A significant difference in the proportion of reads assigned to the Hungate1000 Collection using the RB approach was found between high- and low- methane animals for both *Ape*KI (p = 9.3 × 10^−6^) and *Pst*I (p = 3.4 × 10^−4^; Table 2). This may be attributed to the presence or absence of some species associated with methane yield in the Hungate1000 Collection. For example, Kittelmann et al. [3] identified the genera *Fibrobacter*, *Kandleria*, *Olsenella* and *Sharpea* to be in higher prevalence in low-methane yield animals. These genera are all present within the Hungate1000 Collection and have equal or significantly higher abundance in samples from low-methane animals. The Hungate1000 Collection also has poor or no representation of other genera that were found by Kittelmann et al. [3] to be in higher abundance in high-methane yield animals e.g. *Coprococcus*. This shows that using a method that is reliant on a reference database is limited by the genomes present within the reference database that is used.

**Table 2:** Hit rates by taxonomic level from RE-RRS samples using one of two restriction enzymes.

Restrictionenzyme	Sample^1^	Hit rate by taxonomic level (%)						
		Kingdom	Phylum	Class	Order	Family	Genus	Species
***Ape*KI**	High	20.2	19.4	19.0	19.0	18.0	17.8	4.8
	Low	22.1	21.3	21.0	20.9	19.9	19.7	5.9
***Pst*I**	High	25.3	24.6	24.2	24.2	23.0	22.6	5.8
	Low	27.0	26.0	26.0	26.0	24.7	24.3	6.7

1. Methane yield classification (high- or low-methane yield) of the sheep the sample came from.

A major gap in microbial genome assemblies is the inability, at least historically, to sequence the uncultured microbes that make up a large proportion of any environment [15]. Technological advances, such as single-cell sequencing [34] and the ability to assemble genomes from metagenomic datasets [35], offer alternative solutions to sequence and assemble microbial genomes and will provide opportunities to improve reference databases. Judicious addition of new microbial genome assemblies as they become available will improve hit rates, however, any additional sequences added to the database will also increase the time to complete the analysis, which may not be desirable for a high-throughput approach if there are time constraints. If additional genomes were to be added to the Hungate1000 Collection (or another reference database), expert curation would be needed to ensure the quality of genome assemblies, the accuracy of taxonomic assignment, and to balance the resource across taxa to maximize coverage and minimize duplication.

**Fig 1:**
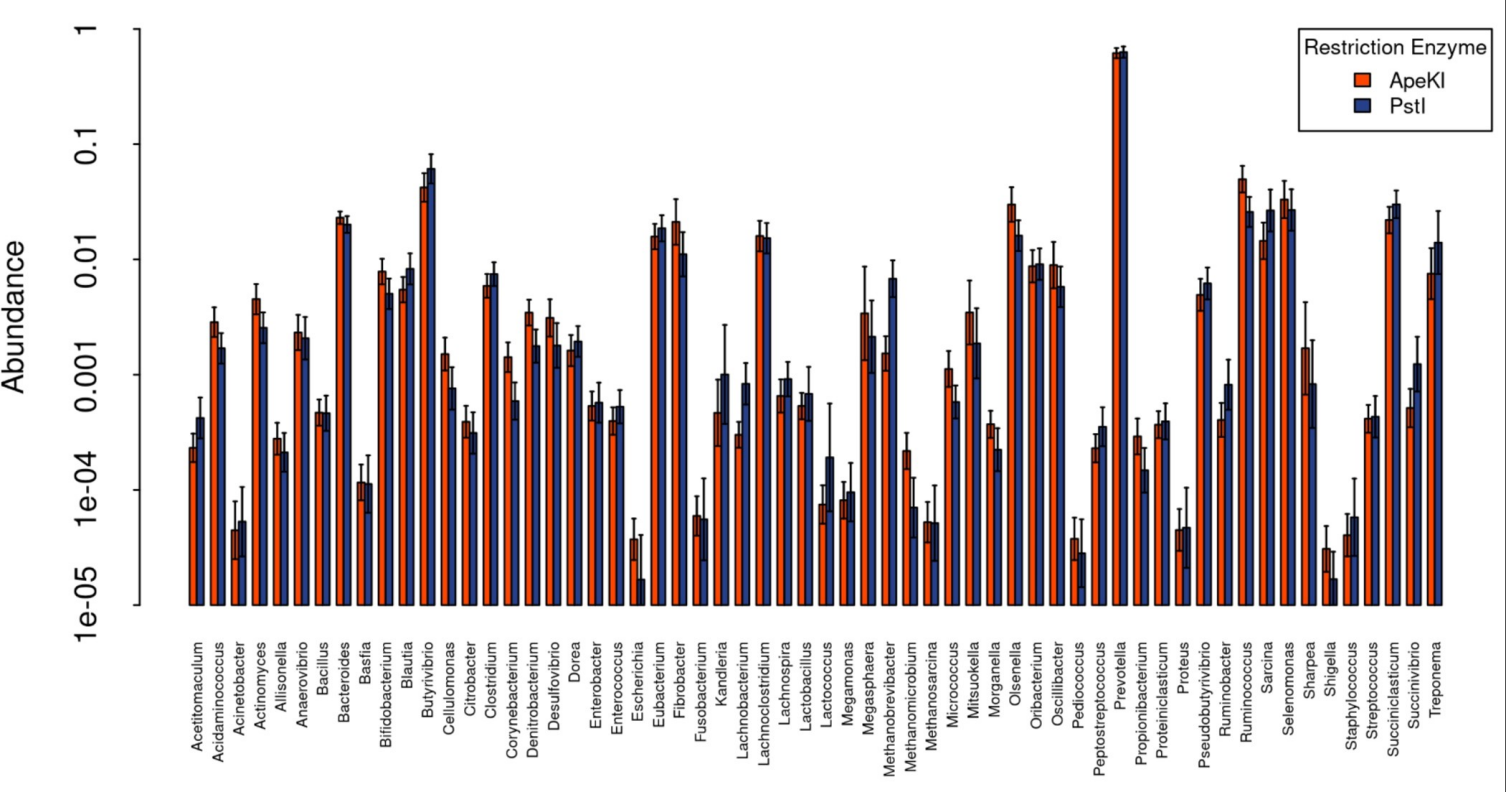
Average abundance (SD) of Hungate1000 Collection genera from the reference-based approach.

The abundance of each genus was very similar between sequences generated using *Ape*KI and *Pst*I (Fig 1). There were a few genera that did show significantly different abundances when using the two restriction enzymes, of note are the methanogens *Methanobrevibacter*, where *Pst*I was significantly more abundant, and *Methanomicrobium*, where *Ape*KI was significantly more abundant. These differences, as well as the other smaller differences observed, can largely be attributed to the proportion of the genome that is captured by each restriction enzyme: the in silico digestion of the Hungate1000 Collection in Hess et al. [22] showed that on average, *Ape*KI captured 29 times the fraction of the genome that digestion by *Pst*I, with *Methanobrevibacter* this was 7 times and for *Methanomicrobium* this was 151 times.

### Reference-free approach

The reference-free approach is not subject to the biases of the species represented in the Hungate1000 Collection. We explored the use of different tag lengths and filtering thresholds and showed that filtering threshold generally had minimal impact on performance–particularly for *Pst*I–until there were very few tags retained (i.e. when tags were required to be present in all samples; S2 File). Tags that were 65 bp long and present in at least 25% of samples were deemed to be the most appropriate for further comparisons because of their generally high repeatability, correlation with methane yield and microbiability (S2 File). Using a filtering threshold that was too high (e.g. 100%) reduced the number of tags to the point where informative tags were removed, while using a threshold that was too low (e.g. 10%) reduced the “signal to noise” ratio such that the repeatability and the methane yield microbiability were reduced.

Using 65 bp reads present in at least 25% of samples resulted in 233,587 *Ape*KI tags that captured 9.9 ± 2.5% of reads, and 502,900 *Pst*I tags that captured 53.3 ± 5.9% of reads. S2 File shows that *Pst*I captures a greater proportion of reads than *Ape*KI at all filtering levels and tag lengths. These differences can be explained by the proportion of each microbial genome that is expected to be captured using each restriction enzyme: *Ape*KI captures 8.6% of Hungate1000 Collection genomes on average, while *Pst*I captures 0.3% [22]. This means that, for a given number of sequences (e.g. one lane of sequencing), the fewer regions captured by *Pst*I reads will be at greater depth, whereas the greater number of regions captured by *Ape*KI will be at lower depth. This is shown by the much larger number of unique tags present when using *Ape*KI compared to *Pst*I (S1 File) despite a slightly larger number of reads per sample for *Pst*I (Table 1).

The reference-free pipeline is particularly useful for prediction of a trait that is correlated with a microbial profile because knowledge of the taxonomic group that a sequence belongs to is of less importance than its predictive ability. Sequences that don’t align to a reference genome can still be used. Given the large number of tags generated using the reference-free approach it may be desirable to cluster these into groups (e.g. through sequence similarity, taxonomic assignment, high positive correlations between tag abundances). If clustered appropriately, this has the potential to add power to analyses; however, if unrelated tags are clustered together, this may weaken the analyses. Tags that come from the same organism will be highly correlated, but a high correlation (positive or negative) could also come about due to interactions between the microbes, or by chance. If taxonomic information is desired, for biological importance or clustering purposes, tags can be searched against a relevant database; this process is computationally inexpensive because there are fewer search terms, i.e. fewer tags (hundreds of thousands) than the full set of reads in the original dataset (tens or hundreds of millions).

### Comparison of tags against the GenBank database

**Fig 2:**
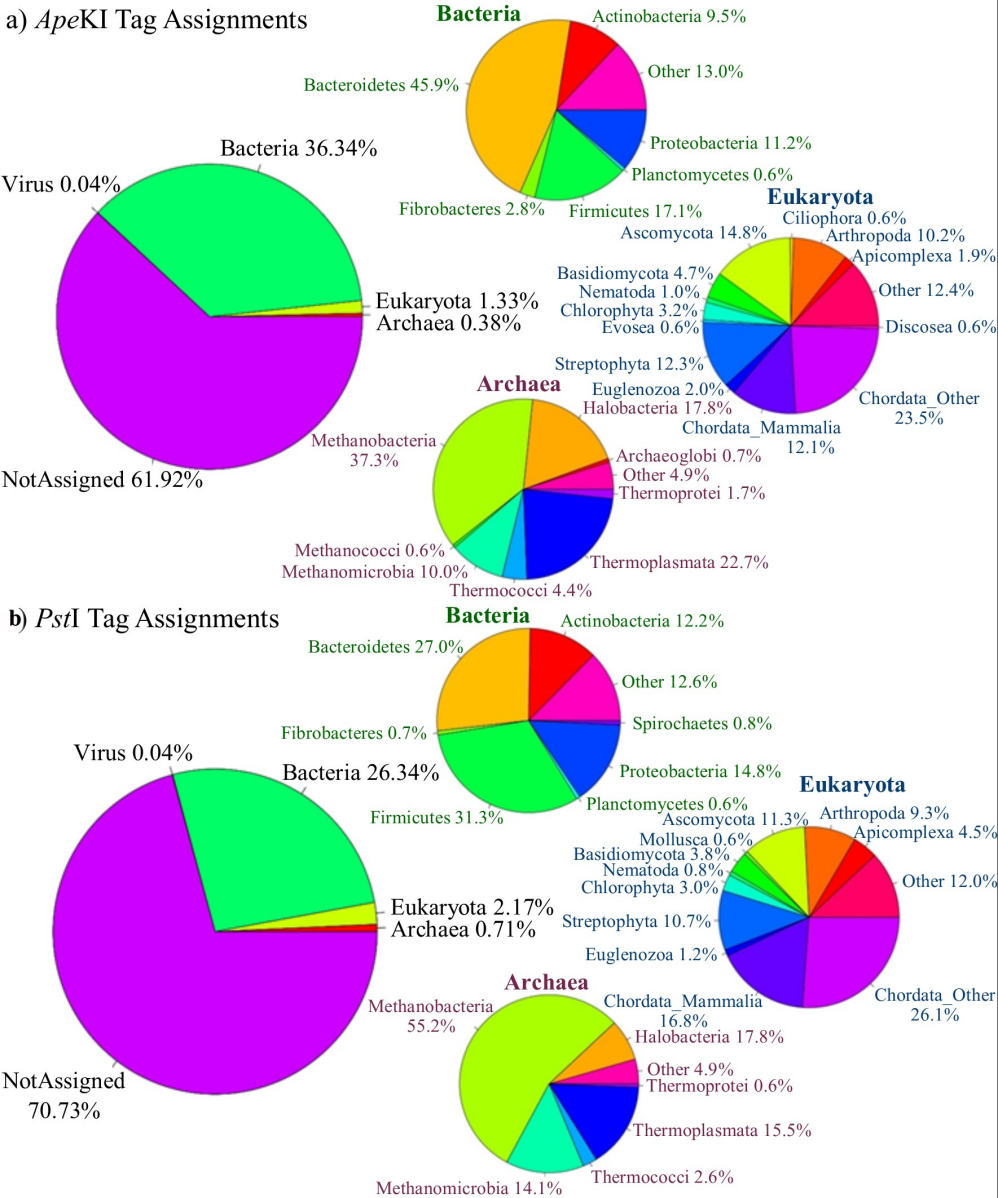
GenBank taxonomies of reference-free tags for *Ape*KI (a) and *Pst*I (b). Tags were compared against the GenBank database using BLAST and taxonomy was assigned using the MEGAN algorithm considering only hits with the top bitscore for that tag. This figure shows the taxonomy of tags at the kingdom level, and within bacteria and eukaryota at the phylum level and within archaea at the class level. Graphs show the proportion of tags assigned to each taxonomic level and do not reflect the relative abundance of each tag.

Taxonomy was assigned to each of the tags by comparing tags to the GenBank database (28)(Fig 2). A large proportion of the reads did not have taxonomy assigned (*Ape*KI: 61.92%, *Pst*I: 70.73%) which is partially due to the absence of genome assemblies in the GenBank database, particularly for uncultured microbes [15], but may also be due to the contamination of sequences in the GenBank database [36], whereby a sequence is assigned to the incorrect organism (e.g. microbial genome incorrectly inserted into a “host” genome assembly)–in these cases the sequence may match to reads in two different kingdoms (one correct, and one incorrect) and therefore be unassigned. Tags were also assigned at a variety of taxonomic levels, suggesting that some tags will represent various microbes within a family, while other tags may capture intra-species variation.

The major groups of rumen microbes that we would expect to see were captured by both restriction enzymes, including the bacterial phyla *Bacteroidetes*, *Fibrobacteres*, *Firmicutes*, *Actinobacteria*, and the methanogen classes *Methanobacteria* and *Methanomicrobia* (Fig 2). The proportion of tags that were present for these groups differed for the two restriction enzymes, but it is important to note that the proportion of tags is not directly related to the abundance of each tag in each sample, just the number of samples each tag is present in and the proportion of each genome that is captured using our RE-RRS approach. *Pst*I (CTGCAG) has a lower GC content than *Ape*KI (GCWGC), therefore the proportion of each genome captured will be related to the GC content of that genome. One example of this is the *Firmicutes* phylum, which generally has a lower GC content than many other bacteria, perhaps explaining why more tags are assigned to *Firmicutes* when using *Ape*KI (Fig 2).

There is a shortage of genome data for many rumen microbes, especially for eukaryotic microbes (e.g. protozoa and fungi), and for some groups of archaea. A BLAST search would find a close match in another group that has a homologue, which could explain the presence of some of the unexpected groups in archaea and eukaryota (e.g. halobacteria are not present within the rumen but are relatives of some methanogen groups; Fig 2). However, microbes that were found to be associated with ruminotype in Kittelmann et al. (3) were identified in the set of tags from each enzyme at either the taxonomic level stated or at one taxonomic level higher. The reason some of them were assigned taxonomy at the level higher could be due to underrepresentation in the GenBank database or the fragment captured by RE-RRS having high sequence similarity to closely related groups.

### Comparison of methods for obtaining RMC profiles

#### Visualization of principal components

**Fig 3:**
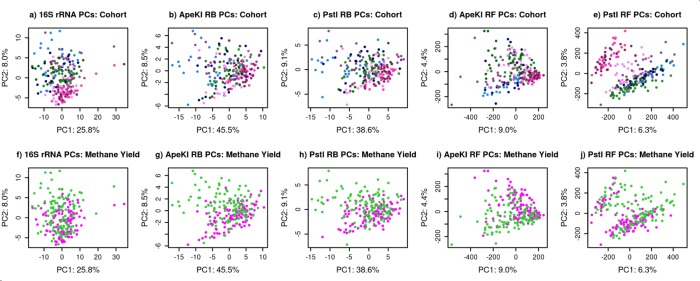
First and second principal components of five metagenome profiling approaches colored by cohort or methane yield. Metagenome Profiling Approaches included 16S rRNA gene sequencing (a and f), and four restriction enzyme reduced representation sequencing approaches: Reference-Based with the *Ape*KI (b and g) and *Pst*I (c and h) restriction enzymes, and Reference-Free with the *Ape*KI (d and i) and PstI (e and j) restriction enzymes. a–e are colored by cohort, with lighter shades of the same color referring to the first sample collected from each sheep and the darker shades referring to the second sample collected from each sheep. f–j are colored by methane yield classification with samples from sheep with low methane yield colored in green and samples from sheep with high methane yield colored in pink.

The first two principal components were plotted against each other to compare major drivers of the profiles for the 16S rRNA gene sequencing and RE-RRS approaches (Fig 3). Although these plots revealed different relationships between samples for each of the groups, cohort and methane yield classification were shown to be major drivers of these relationships for all sequencing approaches. Samples from cohort 1 (shown in blue), which contained only males, tended to cluster together, slightly removed from other cohorts, which contained only females. The sex of the host has been shown to impact RMC profiles [37], which could explain this separation.

### Variance components of RMC profiles

The first and second principal components (PC1 and PC2) were analyzed as a trait for the four RE-RRS approaches and the 16S rRNA gene taxonomic classifications. The percent variance explained by the principal components was negatively correlated with the number of tags or taxa. The reference-based approaches assigned reads to only 60 genera, the 16S rRNA gene approach assigned reads to ~250 genera, and the reference-free approach assigned reads to ~503k and ~523k tags for *Ape*KI and *Pst*I, respectively (Table 3). The greatest percent variance explained by PC1 was for the *Ape*KI_RB approach and was less than 50%, which indicates that a large proportion of the variation in the RMC profile was not accounted for by analyzing only PC1 for these profiles, particularly for the reference-free approach where PC1 explained only 6.3% of the variance in the *Pst*I_RF RMC profile. Nevertheless, evaluating PC1 and PC2 allowed us to easily compare the different approaches and below we discuss statistical approaches that will use more of the information contained within the profile.

**Table 3:** Comparison of metagenome profiling approaches.

Method^1^	Principal Component	PC % Variance^2^	Cohort % Variance^3^	Repeatability^4^	|r_p_ (CH_4_ Yield)|^5^	Microb.^6^
16S rRNA	PC1	25.8	18.7	0.08 (0.10)	0.17 (0.07)	0.19 (0.07)
	PC2	8.0	46.8	0.11 (0.09)	0.48 (0.05)	
*Ape*KI_RB	PC1	45.5	33.5	0.25 (0.09)	0.37 (0.06)	0.28 (0.07)
	PC2	8.5	17.7	0.50 (0.07)	0.51 (0.05)	
*Pst*I_RB	PC1	38.6	31.1	0.24 (0.09)	0.29 (0.06)	0.26 (0.07)
	PC2	9.1	25.3	0.48 (0.07)	0.48 (0.06)	
*Ape*KI_RF	PC1	9.0	42.8	0.28 (0.09)	0.36 (0.06)	0.35 (0.09)
	PC2	4.4	40.2	0.18 (0.09)	0.48 (0.05)	
*Pst*I_RF	PC1	6.3	54.8	0.49 (0.07)	0.43 (0.06)	0.41 (0.08)
	PC2	3.8	52.9	0.50 (0.07)	0.46 (0.06)	

1. 16S rRNA gene sequencing; Restriction Enzyme Reduced Representation Sequencing using *Ape*KI or *Pst*I restriction enzymes and the reference-based (RB) or reference-free (RF) pipelines.

2. Percent of total metagenomic variance explained by PC1 or PC2.

3. Percent of the variance in PC1 or PC2 explained by cohort.

4. Percent of the variation in PC1 and PC2 (after adjusting for cohort) that is due to the permanent environmental effect.

5. Absolute value of the correlation of PC1 and PC2 (after adjusting for cohort) with methane yield.

6. Microbiability: Proportion of the variance in methane yield that can be attributed to the microbial relationship matrix.

Repeatability is a measure of the similarity of two samples from the same individual and was estimated as the proportion of the variance in the principal component (after removal of the cohort effect) that is explained by the permanent environmental effect. The repeatabilities of the principal components for 16S rRNA gene sequences were both lower than any repeatability estimate for the RE-RRS approaches (Table 3). The repeatability of the RE-RRS approaches were consistent, with the repeatability of the first component ~0.25 and the second ~0.50; the exception to this is the lower PC2 estimate for the *Ape*KI_RF approach, and the higher estimate for PC1 for the *Pst*I_RF approach. The high repeatabilities for the *Pst*I_RF approach indicate that this approach is able to capture the portion of the RMC profile that is consistent across time, while the high percent variance attributed to the cohort effect show that this approach is also powerful because the first two components also capture the cohort-specific effects well (Fig 3 and Table 3).

### Microbes and methane yield

The absolute correlation with methane yield was moderate for both principal components for all profiling approaches (Table 3). PC2 had a stronger correlation with methane yield than PC1 for all approaches, and this estimate was consistent across all profiling approaches at ~0.50 (Table 3 and Fig 3). The absolute correlation between PC1 and methane yield was weakest for 16S rRNA gene sequencing and strongest for the *Pst*I_RF approach (Table 3). Correlating PC1 and PC2 with methane yield provides an indication of the amount of variance in methane yield that can be explained by variation in that PC (i.e. only a fraction of the total variance of the RMC profile), the microbiability accounts for the full RMC profile to estimate this. The microbiability estimate was weakest for the 16S rRNA gene sequencing approach and strongest for the *Pst*I_RF approach (Table 3). The microbiability estimates were similar for the two reference-based approaches and the *Ape*KI_RF approach was intermediate between the reference-based approaches and the *Pst*I_RF approach.

Together with the repeatability results, these results indicate that the RE-RRS approaches will be at least as good as 16S rRNA gene sequencing for capturing the RMC profiles (Table 3). Overall, the results using the *Pst*I restriction enzyme were better than the results using the *Ape*KI restriction enzyme, and the results for the reference-free pipeline were better than the reference-based. These results suggest that our RE-RRS approach will predict the individual’s methane production better than 16S rRNA gene sequencing, but more samples are needed to evaluate whether reducing methane emissions by selection of individuals based on their RMC profile will translate to offspring that also have reduced methane emissions.

The stronger relationships between methane yield and the reference-free approaches (Table 3) suggest that these approaches might be capturing components of the rumen microbiome that are not being captured by bacterial 16S rRNA gene sequencing or the reference-based approaches. The 16S rRNA gene method only targeted bacteria, while the reference-based approach focused on capturing bacterial and archaeal community profiles but were limited to the reference database being used. The reference-free approach is capturing DNA from a much wider taxonomic range, e.g. host, feed, protozoa, fungi and viruses (Fig 2) that are absent from the Hungate1000 Collection. If the aim is to obtain the most accurate predictions, then this information from a wider taxonomic range is expected to be beneficial to include in the analysis. The performance of the reference-based approach could further be improved by generating a more comprehensive genome database.

### Sensitivity to sequencing depth

**Fig 4:**
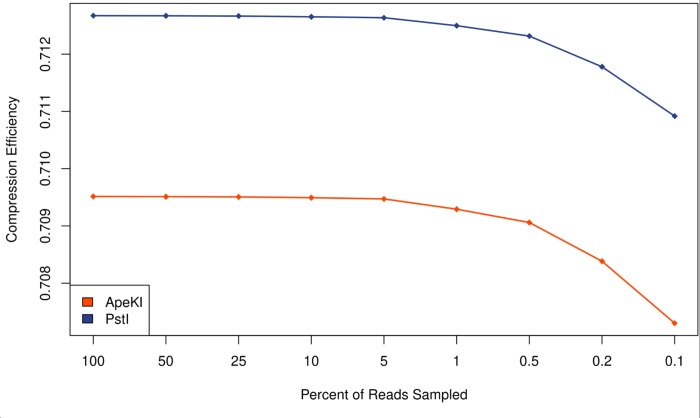
Compression efficiency of RE-RRS data as the percent of reads sampled decreases. The compression efficiency of sequence data decreases when less than 5% of reads are sampled, with a sequencing depth that corresponds to 20 times the number of samples sequenced per lane. This number was consistent for both restriction enzymes (*Ape*KI and *Pst*I) used for this study. Standard errors were 0.000 and are therefore not shown.

The number of samples per lane influences the cost per sample of sequencing as well as the average number of sequenced reads per sample. A sensitivity analysis was performed by subsampling reads from our RE-RRS samples and evaluating the compression efficiency of the dataset. This showed that sampling 5% of reads, corresponding to 20 times the number of samples per lane, i.e. 2000 samples, is the lower bound because the compression efficiency begins to drop (Fig 4).

*Ape*KI had a lower compression efficiency than *Pst*I (Fig 4) because *Ape*KI captures more regions of the microbial genome at lower depth than *Pst*I. It is therefore not as powerful for the reference-free approach. By extension, the reference-free approach developed here would not be suitable for metagenome shotgun sequencing data (unless the sequencing depth was extremely high), because any part of the metagenome could be captured. This could explain the poor results using a k-mer approach in Ross et al. [38]. Therefore, the profiling pipeline needs to be chosen based on the sequencing approach used and the intended analysis.

We determined a potential cut-off for how much we can increase the throughput without losing crucial information, but it should be noted that these samples are from a relatively small set of individuals that have extreme phenotypes. More sequences may be needed when the spread of methane yields is more continuous, but this remains to be tested. This analysis shows that the depth of sequencing used in this study was well within reasonable bounds for capturing metagenomics data. We suggest that throughput could be safely increased 2–4× over what was done in this study by correspondingly reducing the sequencing depth per sample. This will reduce costs and allow faster turn-around times for obtaining sequencing data when large numbers of samples are analyzed. RE-RRS using *Pst*I as described here with 384 samples per lane would result in a similar cost-point for RE-RRS and 16S rRNA gene sequencing and will be used for future studies.

### Utility of a high-throughput metagenomics method

#### RE-RRS vs. 16S rRNA gene sequencing and metagenome shotgun sequencing

Our RE-RRS approach to sequencing rumen samples is likely to perform as well or better than 16S rRNA gene sequencing in terms of the variation in sequence reads that is accounted for, and the predictive ability of RMC profiles (Table 3). Although RE-RRS can capture taxonomic information, like 16S rRNA gene sequencing it cannot directly quantify the abundance of particular genes within a sample, and most genes will be missed because it is a reduced representation sequencing approach that only captures a small percentage of each microbial genome and is directed at certain genomic regions due to the use of restriction endonucleases. Metagenome shotgun sequencing can capture information on the relative abundances of these genes in addition to taxonomic information. However, the quantity of sequence required is 10- to 100-fold greater, which is very expensive and takes considerable time to analyze.

Reduced representation sequencing of metagenome samples using restriction enzyme has previously been shown to perform well compared to 16S and metagenome shotgun sequencing [39–41]. While our study used a single digest of *Ape*KI or *Pst*I, these studies all used a double digest (i.e. digestion of the DNA by two different restriction enzymes) and they did not show the utilization of this approach as a high-throughput sequencing approach to the extent that we have, with the number of samples analyzed ranging from 3 to 17 and samples sequenced at much higher depth. In addition, the approach described in Liu et al. [41] focused on only a reference-based approach and did not explore the potential of a reference-free approach.

#### Other sample types

Most metagenome studies in livestock have used small sample sizes and many used animals with extreme phenotypes, which is valuable for identifying whether there is a relationship between microbes and traits of interest. However, knowledge of the RMC of thousands of animals has the potential to reduce the carbon footprint of farming through selection of individuals with a rumen microbiome genetically associated with lower methane emissions. Traits aimed at reducing the carbon footprint of livestock animals, e.g. methane emissions or feed efficiency, are often difficult and expensive to measure. Therefore, provided the costs can be reduced sufficiently and high-throughput profiling is possible, large volumes of samples can be processed and the data analyzed quickly and cheaply. In this situation, metagenome profiling could provide an alternative solution to reducing the carbon footprint that circumvents the need to continually measure expensive methane yield phenotypes on thousands of animals.

Much research has been done into sequencing microbial samples from humans [42, 43], particularly samples related to the digestive tract and their association with a variety of health issues [1, 2]. A low-cost, high-throughput metagenome sequencing approach has the potential to make screening of these samples more accessible to those that require them. High-throughput metagenome profiling has the potential to improve monitoring of other environmental samples as well. This could range from identifying pathogens in water samples, evaluating the quality of water in different environments, to identifying favorable and unfavorable soil environments for the crop growth. Further research is required to evaluate the potential of RE-RRS in each of these situations.

## Conclusions

We have shown that RE-RRS is a promising method for obtaining low-cost, high-throughput metagenomic profiles and performs at least as well as bacterial 16S rRNA gene sequencing. Metagenomic profiles can be generated either with or without a reference database (reference-based or reference-free, respectively) depending on the purpose of the analysis. Gathering metagenomic information on a large number of animals can be a useful addition to genomic information for the prediction of traits in livestock production and human health. The next steps are to use this approach to sequence thousands of environmental samples and develop appropriate statistical models for prediction purposes.

## Supporting information

S1 Table Sample and sequencing information. This file contains information on the samples, including associated animal information and methane yield phenotypes, sequencing accession numbers for the 16S rRNA gene sequences and the RE-RRS sequences, as well as information on sample trimming for RE-RRS, assignment rates for RB and RF approaches and the percent of sequences that align to the host genome.
(XLSX)

S1 Fig Sequence quality per base pair for all lanes of sequencing. Box and whisker plots of sequence quality (Phred Score) at positions along the sequenced read. Red, orange and green signify low, medium and high-quality bases, respectively. Sequence quality was high throughout the entire read, however it did drop slightly towards the end of the read. Sequence quality for *Ape*KI was more variable than for *Pst*I. The third plot for *Pst*I represents the 94 samples that were re-sequenced due to barcodes not ligating in the initial run.
(TIF)

S1 File Comparison of BLAST parameters. This file contains an investigation into suitable BLASTN parameters for use in the reference-based RE-RRS approach.
(DOCX)

S2 File Tag filtering comparison. This file contains an investigation into suitable tag lengths and tag prevalence thresholds for use with *Ape*KI and *Pst*I restriction enzymes.
(DOCX)

## References

[1] Young W, Jester T, Stoll ML, Izcue A. Inflammatory Bowel Disease In: Ragab G, Atkinson T, Stoll M, editors. The Microbiome in Rheumatic Disease and Infection. Cham: Springer; 2018.

[2] Lebwohl B, Sanders DS, Green PH. Coeliac disease. The Lancet. 2018;391(10115):70–81. 10.1016/S0140-6736(17)31796-8 28760445

[3] Kittelmann S, Pinares-Patiño CS, Seedorf H, Kirk MR, Ganesh S, McEwan JC, et al Two different bacterial community types are linked with the low-methane emission trait in sheep. PLOS ONE. 2014;9(7):e103171 10.1371/journal.pone.0103171 25078564 PMC4117531

[4] Difford GF, Plichta DR, Løvendahl P, Lassen J, Noel SJ, Højberg O, et al Host genetics and the rumen microbiome jointly associate with methane emissions in dairy cows. PLOS Genet. 2018;14(10):e1007580 10.1371/journal.pgen.1007580 30312316 PMC6200390

[5] Shabat SKB, Sasson G, Doron-Faigenboim A, Durman T, Yaacoby S, Miller MEB, et al Specific microbiome-dependent mechanisms underlie the energy harvest efficiency of ruminants. ISME J. 2016;10(12):2958 10.1038/ismej.2016.62 27152936 PMC5148187

[6] Sasson G, Ben-Shabat SK, Seroussi E, Doron-Faigenboim A, Shterzer N, Yaacoby S, et al Heritable bovine rumen bacteria are phylogenetically related and correlated with the cow’s capacity to harvest energy from its feed. MBio. 2017;8(4):e00703–17. 10.1128/mBio.00703-17 28811339 PMC5559629

[7] Shi W, Moon CD, Leahy SC, Kang D, Froula J, Kittelmann S, et al Methane yield phenotypes linked to differential gene expression in the sheep rumen microbiome. Genome Res. 2014;24(9):1517–25. 10.1101/gr.168245.113 24907284 PMC4158751

[8] Kamke J, Kittelmann S, Soni P, Li Y, Tavendale M, Ganesh S, et al Rumen metagenome and metatranscriptome analyses of low methane yield sheep reveals a Sharpea-enriched microbiome characterised by lactic acid formation and utilisation. Microbiome. 2016;4(1):56 10.1186/s40168-016-0201-2 27760570 PMC5069950

[9] Rowe SJ, Kittelmann S, Pinares-Patiño CS, Wood G, Dodds KG, Kirk MR, et al, editors. BRIEF COMMUNICATION: Genetic control of the rumen microbiome in sheep. Proceedings of the New Zealand Society of Animal Production; 2015.

[10] DeSantis TZ, Hugenholtz P, Larsen N, Rojas M, Brodie EL, Keller K, et al Greengenes, a chimera-checked 16S rRNA gene database and workbench compatible with ARB. Appl. Environ. Microbiol. 2006;72(7):5069–72. 10.1128/AEM.03006-05 16820507 PMC1489311

[11] Henderson G, Yilmaz P, Kumar S, Forster RJ, Kelly WJ, Leahy SC, et al Improved taxonomic assignment of rumen bacterial 16S rRNA sequences using a revised SILVA taxonomic framework. PeerJ. 2019;7:e6496 10.7717/peerj.6496 30863673 PMC6407505

[12] Franzén O, Hu J, Bao X, Itzkowitz SH, Peter I, Bashir A. Improved OTU-picking using long-read 16S rRNA gene amplicon sequencing and generic hierarchical clustering. Microbiome. 2015;3(1):43. 26434730 10.1186/s40168-015-0105-6 PMC4593230

[13] Sim K, Cox MJ, Wopereis H, Martin R, Knol J, Li M-S, et al Improved detection of bifidobacteria with optimised 16S rRNA-gene based pyrosequencing. PLOS ONE. 2012;7(3):e32543 10.1371/journal.pone.0032543 22470420 PMC3314643

[14] Seshadri R, Leahy SC, Attwood GT, Teh KH, Lambie SC, Cookson AL, et al Cultivation and sequencing of rumen microbiome members from the Hungate1000 Collection. Nat. Biotechnol. 2018;36(4):359 10.1038/nbt.4110 29553575 PMC6118326

[15] Fodor AA, DeSantis TZ, Wylie KM, Badger JH, Ye Y, Hepburn T, et al The “most wanted” taxa from the human microbiome for whole genome sequencing. PLOS ONE. 2012;7(7). 10.1371/journal.pone.0041294 PMC3406062 22848458

[16] Elshire RJ, Glaubitz JC, Sun Q, Poland JA, Kawamoto K, Buckler ES, et al A robust, simple genotyping-by-sequencing (GBS) approach for high diversity species. PLOS ONE. 2011;6(5):e19379 10.1371/journal.pone.0019379 21573248 PMC3087801

[17] Dussex N, Taylor HR, Stovall WR, Rutherford K, Dodds KG, Clarke SM, et al Reduced representation sequencing detects only subtle regional structure in a heavily exploited and rapidly recolonizing marine mammal species. Ecol Evol. 2018;8(17):8736–49. 10.1002/ece3.4411 30271541 PMC6157699

[18] Pinares-Patiño C, Hickey S, Young E, Dodds K, MacLean S, Molano G, et al Heritability estimates of methane emissions from sheep. Animal. 2013;7(s2):316–21. 10.1017/S1751731113000864 PMC3691003 23739473

[19] Caporaso JG, Kuczynski J, Stombaugh J, Bittinger K, Bushman FD, Costello EK, et al QIIME allows analysis of high-throughput community sequencing data. Nat Methods. 2010;7(5):335 10.1038/nmeth.f.303 20383131 PMC3156573

[20] Andrews S. FastQC: a quality control tool for high throughput sequence data. Babraham Bioinformatics, Babraham Institute, Cambridge, United Kingdom; 2010.

[21] Bragg L, Stone G, Imelfort M, Hugenholtz P, Tyson GW. Fast, accurate error-correction of amplicon pyrosequences using Acacia. Nat Methods. 2012;9(5):425 10.1038/nmeth.1990 22543370

[22] Hess MK, Rowe SJ, Van Stijn TC, Brauning R, Hess AS, Kirk MR, et al High-throughput rumen microbial profiling using genotyping-by-sequencing. World Congress for Genetics Applied to Livestock Production; Auckland, New Zealand 2018.

[23] Herten K, Hestand MS, Vermeesch JR, Van Houdt JK. GBSX: a toolkit for experimental design and demultiplexing genotyping by sequencing experiments. BMC Bioinformatics. 2015;16(1):73. 25887893 10.1186/s12859-015-0514-3 PMC4359581

[24] Krueger F. Trim Galore: A wrapper tool around Cutadapt and FastQC to consistently apply quality and adapter trimming to FastQ files. 2015.

[25] Li H. Aligning sequence reads, clone sequences and assembly contigs with BWA-MEM. arXiv preprint arXiv:13033997. 2013.

[26] Camacho C, Coulouris G, Avagyan V, Ma N, Papadopoulos J, Bealer K, et al BLAST+: architecture and applications. BMC Bioinformatics. 2009;10(1):421. 20003500 10.1186/1471-2105-10-421 PMC2803857

[27] Huson DH, Auch AF, Qi J, Schuster SC. MEGAN analysis of metagenomic data. Genome Res. 2007;17(3):377–86. 10.1101/gr.5969107 17255551 PMC1800929

[28] Sayers EW, Cavanaugh M, Clark K, Ostell J, Pruitt KD, Karsch-Mizrachi I. GenBank. Nucleic Acids Res. 2019;47(D1):D94–D9. 10.1093/nar/gky989 30365038 PMC6323954

[29] Gilmour A, Gogel B, Cullis B, Welham S, Thompson R. ASReml user guide release 4.1 structural specification. Hemel Hempstead: VSN International Ltd 2015.

[30] Ross E, Moate P, Marett L, Cocks B, Hayes B. Investigating the effect of two methane-mitigating diets on the rumen microbiome using massively parallel sequencing. J Dairy Sci. 2013;96(9):6030–46. 10.3168/jds.2013-6766 23871375

[31] Hudson NJ, Porto-Neto LR, Kijas J, McWilliam S, Taft RJ, Reverter A. Information compression exploits patterns of genome composition to discriminate populations and highlight regions of evolutionary interest. BMC Bioinformatics. 2014;15(1):66. 24606587 10.1186/1471-2105-15-66 PMC4015654

[32] Gailly J-l. gzip. 1.3.12 ed. http://www.gzip.org/2007

[33] Ziv J, Lempel A. A universal algorithm for sequential data compression. IEEE Trans Inf Theory. 1977;23(3):337–43.

[34] Hwang B, Lee JH, Bang D. Single-cell RNA sequencing technologies and bioinformatics pipelines. Exp Mol Med. 2018;50(8):96 10.1038/s12276-018-0071-8 30089861 PMC6082860

[35] Parks DH, Rinke C, Chuvochina M, Chaumeil P-A, Woodcroft BJ, Evans PN, et al Recovery of nearly 8,000 metagenome-assembled genomes substantially expands the tree of life. Nat Microbiol. 2017;2(11):1533 10.1038/s41564-017-0012-7 28894102

[36] Steinegger M, Salzberg SL. Terminating contamination: large-scale search identifies more than 2,000,000 contaminated entries in GenBank. bioRxiv. 2020. 10.1186/s13059-020-02023-1 PMC7218494 32398145

[37] Cunningham HC, Austin KJ, Cammack KM. Influence of maternal factors on the rumen microbiome and subsequent host performance. Transl Anim Sci. 2018;2(suppl_1):S101–S5. 10.1093/tas/txy058 PMC7200922 32704752

[38] Ross EM, Moate PJ, Marett LC, Cocks BG, Hayes BJ. Metagenomic predictions: from microbiome to complex health and environmental phenotypes in humans and cattle. PLOS ONE. 2013;8(9):e73056 10.1371/journal.pone.0073056 24023808 PMC3762846

[39] Avershina E, Angell IL, Simpson M, Storrø O, Øien T, Johnsen R, et al Low maternal microbiota sharing across gut, breast milk and vagina, as revealed by 16S rRNA gene and reduced metagenomic sequencing. Genes. 2018;9(5):231. 10.3390/genes9050231 PMC5977171 29724017

[40] Ravi A, Avershina E, Angell IL, Ludvigsen J, Manohar P, Padmanaban S, et al Comparison of reduced metagenome and 16S rRNA gene sequencing for determination of genetic diversity and mother-child overlap of the gut associated microbiota. J Microbiol Methods. 2018;149:44–52. 10.1016/j.mimet.2018.02.016 29501688

[41] Liu MY, Worden P, Monahan LG, DeMaere MZ, Burke CM, Djordjevic SP, et al Evaluation of ddRADseq for reduced representation metagenome sequencing. PeerJ. 2017;5:e3837 10.7717/peerj.3837 28948110 PMC5609526

[42] Methé BA, Nelson KE, Pop M, Creasy HH, Giglio MG, Huttenhower C, et al A framework for human microbiome research. Nature. 2012;486(7402):215 10.1038/nature11209 22699610 PMC3377744

[43] Qin J, Li R, Raes J, Arumugam M, Burgdorf KS, Manichanh C, et al A human gut microbial gene catalogue established by metagenomic sequencing. Nature. 2010;464(7285):59 10.1038/nature08821 20203603 PMC3779803

