# Supplementary figures and images for: A restriction enzyme reduced representation sequencing approach for low-cost, high-throughput metagenome profiling
Source: PLoS One. 2020 Apr 3;15(4):e0219882. doi: 10.1371/journal.pone.0219882 (PMC7122713; doi:10.1371/journal.pone.0219882)

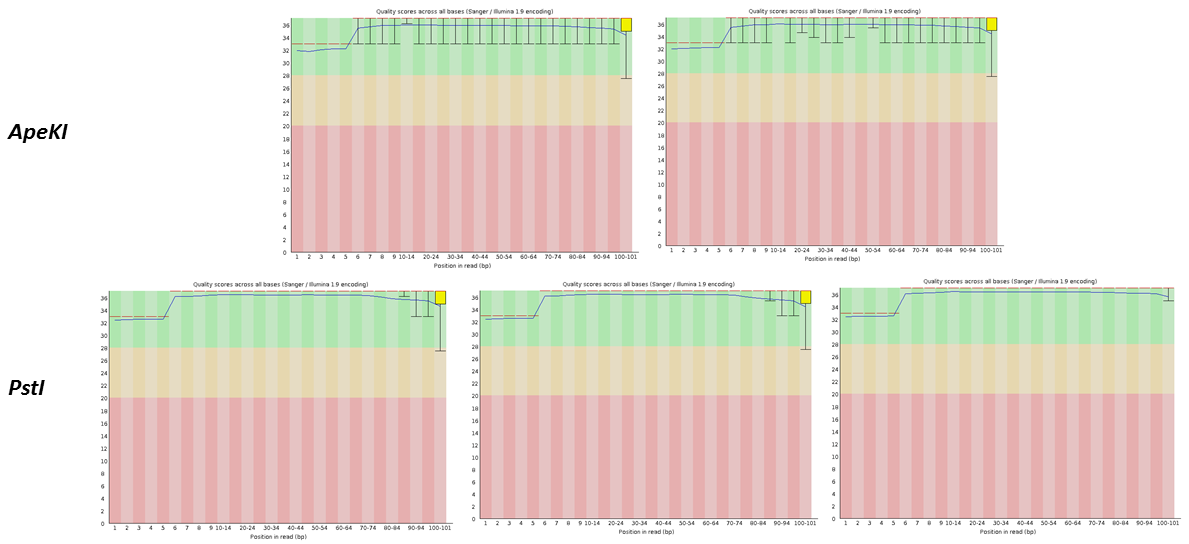

Supplement: S1 Fig — Box and whisker plots of sequence quality (Phred Score) at positions along the sequenced read. Red, orange and green signify low, medium and high-quality bases, respectively. Sequence quality was high throughout the entire read, however it did drop slightly towards the end of the read. Sequence quality for ApeKI was more variable than for PstI. The third plot for PstI represents the 94 samples that were re-sequenced due to barcodes not ligating in the initial run. (TIF) [file pone.0219882.s002.tif]
