# Supplementary material for: A restriction enzyme reduced representation sequencing approach for low-cost, high-throughput metagenome profiling
Source: PLoS One. 2020 Apr 3;15(4):e0219882. doi: 10.1371/journal.pone.0219882 (PMC7122713; doi:10.1371/journal.pone.0219882)
Supplement: S1 File — This file contains an investigation into suitable BLASTN parameters for use in the reference-based RE-RRS approach. (DOCX) [file pone.0219882.s003.docx]

**File S1: Comparison of BLAST Parameters**

**Aim**

Evaluate different Nucleotide BLAST parameters to select appropriate parameters for the reference based pipeline.

**Methods**

*Virtually Digested Reads*

Nucleotide BLAST (Camacho et al., 2009) was used to compare the virtually digested Hungate 1000 Collection reads from Hess et al. (2018) that were a suitable length for GBS against the Hungate 1000 Collection, ignoring the perfect match that the digested read originated from. The BLASTN algorithms that were evaluated were MegaBLAST, BLASTN and Discontiguous MegaBLAST (DC-Mega), with word sizes 11, 16 and 28, as appropriate for each algorithm. Algorithms were compared based on the percent of reads assigned at each taxonomic level (assignment based on the MEGAN algorithm, as described in the main text), and the accuracy of those assignments. Runtime was also taken into consideration.

*Assignment Rates*

The assignment rates using four BLAST algorithms was evaluated for the *Pst*I sequences from the 236 sheep rumen samples. Taxonomies were assigned based on the MEGAN algorithm described in the main text, and algorithms were compared based on the percent of reads assigned at each taxonomic level. Runtime for the BLAST algorithm was also taken into consideration.

*Principal Component Analyses and Microbiability*

The two top BLAST algorithms were compared using the first two components of a principal component analysis and the methane yield microbiability, as described in the main paper.

**Results**

*Virtually Digested Reads*

The percent of reads assigned at each taxonomic level increased as the word size decreased (Table S1.1), however there was also a slight decrease in the accuracy of those assignments and increase in runtime (Table S1.2). The accuracy of all algorithms was greater than 95% at the genus level. DC-Mega had the longest runtime and did not perform better than either of the other algorithms run at the same word size. BLASTN had a slightly higher assignment rate than MegaBLAST for both word size 11 and 16, however the accuracy of MegaBLAST was slightly higher. Based on these results, it was decided that BLASTN with word sizes 11 and 16, and MegaBLAST with word sizes 16 and 28 would be tested on the full RE-RRS dataset.

*Assignment Rates*

The runtimes of the four algorithms evaluated ranged from half an hour to 31 hours, and increased as the word size increased (Table 3). As observed in the virtually digested reads (Table 1), The assignment rate was higher as the word size got smaller, and was higher for BLASTN than MegaBLAST when a word size of 16 was used. Although there is a substantial jump in assignment rate from BLASTN with word size 16 to word size of 11, there is also a very large increase in runtime. Further analyses will be performed on the two BLASTN algorithms, with word size of 11 and 16 to determine whether the ~7% increase in assignment rate when using word size of is providing much information beyond the word size of 16. The two BLASTN options were chosen rather than MegaBLAST with word size of 16 because of the increase in assignment rate with relatively small increase in runtime.

Table S1.1: Percent of Reads Assigned at Each Taxonomic Level when BLASTing Virtually Digested Hungate Reads Against the Hungate Database

| WordSize | BLAST algorithm | Percent VD Hungate Reads Assigned | | | | | | |
| --- | --- | --- | --- | --- | --- | --- | --- | --- |
|  |  | Kingdom | Phylum | Class | Order | Family | Genus | Species |
| 28 | MegaBLAST | 48.9% | 48.9% | 48.9% | 48.8% | 47.5% | 41.1% | 19.9% |
| 16 | MegaBLAST | 65.3% | 65.2% | 65.0% | 64.8% | 62.3% | 52.8% | 25.4% |
| 16 | BLASTN | 67.0% | 66.8% | 66.6% | 66.4% | 63.6% | 53.7% | 25.4% |
| 11 | MegaBLAST | 70.9% | 70.7% | 70.4% | 70.2% | 66.9% | 56.2% | 27.2% |
| 11 | BLASTN | 74.4% | 74.0% | 73.6% | 73.3% | 69.3% | 57.8% | 27.6% |
| 11 | DC-Mega | 71.6% | 71.4% | 71.1% | 70.9% | 67.2% | 56.2% | 26.8% |

Table S1.2: Accuracy of Reads at Each Taxonomic Level when BLASTing Virtually Digested Hungate Reads Against the Hungate Database

| WordSize | BLAST algorithm | Time (m:s) | Accuracy of Assignment | | | | | | |
| --- | --- | --- | --- | --- | --- | --- | --- | --- | --- |
|  |  |  | Kingdom | Phylum | Class | Order | Family | Genus | Species |
| 28 | MegaBLAST | 04:24 | 100.0% | 99.9% | 99.8% | 99.6% | 99.6% | 97.7% | 89.3% |
| 16 | MegaBLAST | 09:36 | 100.0% | 99.6% | 99.4% | 99.0% | 96.6% | 95.9% | 84.4% |
| 16 | BLASTN | 09:51 | 99.9% | 99.5% | 99.1% | 98.8% | 96.1% | 95.5% | 84.0% |
| 11 | MegaBLAST | 57:05 | 100.0% | 99.7% | 99.4% | 99.0% | 96.4% | 95.9% | 83.4% |
| 11 | BLASTN | 34:27 | 100.0% | 99.5% | 99.2% | 98.7% | 95.9% | 95.4% | 82.5% |
| 11 | DC-Mega | 106 | 100.0% | 99.6% | 99.3% | 99.0% | 96.3% | 95.8% | 83.2% |

Table 3: Percent of Reads Assigned at Each Taxonomic Level when BLASTing *Pst*I Samples against the Hungate Database

| WordSize | BLAST algorithm | Time (h) | Percent Sample Reads Assigned | | | | | | |
| --- | --- | --- | --- | --- | --- | --- | --- | --- | --- |
|  |  |  | Kingdom | Phylum | Class | Order | Family | Genus | Species |
| 28 | MegaBLAST | 0.5 | 6.9 ± 1.8% | 6.9 ± 1.8% | 6.9 ± 1.8% | 6.9 ± 1.8% | 6.8 ± 1.8% | 6.8 ± 1.8% | 2.0 ± 1.1% |
| 16 | MegaBLAST | 4 | 23.2 ± 3.4% | 22.7 ± 3.5% | 22.5 ± 3.5% | 22.4 ± 3.5% | 21.5 ± 3.5% | 21.2 ± 3.5% | 5.7 ± 1.3% |
| 16 | BLASTN | 6 | 26.1 ± 3.6% | 25.4 ± 3.6% | 25.1 ± 3.7% | 25.1 ± 3.7% | 23.8 ± 3.7% | 23.5 ± 3.7% | 6.2 ± 1.3% |
| 11 | BLASTN | 31 | 35.8 ±  4.0% | 34.3 ± 4.1% | 33.7 ± 4.2% | 33.6 ± 4.2% | 31.0 ± 4.2% | 30.4 ± 4.2% | 7.5 ± 1.3% |

*Principal Component Analyses*

The principal component (PC) analyses for BLASTN with word sizes 11 and 16 gave similar results, and both analyses show clustering by cohort present in the first and second principal components (Table 4). The percent variance explained by the first and second components was similar for each word size, and each provided similar repeatability estimates and similar correlations with methane yield. The second principal component (PC2) had a higher repeatability and a higher correlation with methane yield than the first principal component (PC1).

Table 4: Variance, Repeatability and Correlations with Methane Yield for BLASTN approaches

| **Method**^1^ | **PC % Variance^2^** | **Cohort % Variance^3^** | **Repeatability^4^** | **\|r_p_ (CH_4_ Yield)\|^5^** | **Microbiability^6^** |
| --- | --- | --- | --- | --- | --- |
| PC1_BN11 | 40.0 | 33.6 | 0.23 ± 0.09 | 0.29 ± 0.06 | 0.28 (0.07) |
| PC2_BN11 | 9.3 | 26.0 | 0.49 ± 0.07 | 0.51 ± 0.05 |  |
| PC1_BN16 | 38.6 | 31.1 | 0.24 ± 0.09 | 0.29 ± 0.06 | 0.26 (0.07) |
| PC2_BN16 | 9.1 | 25.3 | 0.48 ± 0.07 | 0.48 ± 0.06 |  |

1. Principal components 1 or 2, for BLASTN with word size of 11 or 16
2. Percent of total metagenomic variance explained by PC1 or PC2
3. Percent of the variance in PC1 or PC2 explained by cohort
4. Percent of the variation in PC1 and PC2 (after adjusting for cohort) that is due to the permanent environmental effect
5. Absolute value of the correlation of PC1 and PC2 (after adjusting for cohort) with methane yield.
6. Microbiability: Proportion of the variance in methane yield that can be attributed to the microbial relationship matrix

*Microbiability*

The variance of methane yield that can be attributed to the microbiome profile is significantly different from zero and is consistent between the two different word sizes when running a BLASTN algorithm.

**Conclusions**

The BLAST algorithm parameters used were shown to have a large impact on the proportion of reads assigned to each taxonomic level and the runtime of the algorithm, with a minor impact on the accuracy of those assignments. The results for repeatability, correlation with methane yield and microbiability were very similar when comparing BLASTN with a word size of 11 and 16, suggesting there is no benefit to the vastly increased runtime for BLASTN with word size of 11. BLASTN with word size 16 was deemed to be the most appropriate BLAST algorithm for our high-throughput analysis pipeline due to its much faster runtime and similar results to BLASTN with a word size of 11.

**References**

Camacho, C., Coulouris, G., Avagyan, V., Ma, N., Papadopoulos, J., Bealer, K., & Madden, T. L. (2009). BLAST+: architecture and applications. *Bmc Bioinformatics, 10*(1), 421.

Hess, M. K., Rowe, S. J., Van Stijn, T. C., Brauning, R., Hess, A. S., Kirk, M. R., . . . McEwan, J. C. (2018). *High-throughput rumen microbial profiling using genotyping-by-sequencing*. Paper presented at the World Congress for Genetics Applied to Livestock Production, Auckland, New Zealand.
