# Supplementary material for: A restriction enzyme reduced representation sequencing approach for low-cost, high-throughput metagenome profiling
Source: PLoS One. 2020 Apr 3;15(4):e0219882. doi: 10.1371/journal.pone.0219882 (PMC7122713; doi:10.1371/journal.pone.0219882)
Supplement: S2 File — This file contains an investigation into suitable tag lengths and tag prevalence thresholds for use with ApeKI and PstI restriction enzymes. (DOCX) [file pone.0219882.s004.docx]

**File S2: Comparison of Tag Filters**

**Methods**

*Tag Length and Prevalence*

We explored the impact of three tag lengths: 65 bp, 32 bp and 16 bp. All tags began with the cut site and trimmed at the opposite end. The cut-off for inclusion of a tag in the RMC profile (i.e. the count matrix) was evaluated at four thresholds: present in 10%, 25%, 50% or 100% (i.e. all) of samples.

*Performance*

The performance when using different tag lengths and tag prevalence was evaluated using seven metrics: number of tags, percent of reads captured, percent variance explained by the first and second principal components, the percent of the variance in PC1 and PC2 that was explained by cohort, the repeatability of PC1 and PC2, the correlation of PC1 and PC2 with methane yield, and the methane yield microbiability, defined as the proportion of the variance of methane yield that could be attributed to the microbial relationship matrix. Principal components and the microbial relationship matrix were generated and analysed as described in the main text.

**Results**

*Number of Tags and Percent of Reads Captured*

More unique tags were present for *Ape*KI than *Pst*I for all tag lengths, consistent with *Ape*KI capturing a greater proportion of microbial genomes than *Pst*I (Tables S1.1, S1.2). A similar number of unique 65 bp tags were present in only one sample: 66% for *Ape*KI and 68% for *Pst*I.

Within each tag length, when the prevalence filter increases, fewer tags are included and the more each component of the correspondence analysis explains (Tables S1.1, S1.2). When evaluating *Ape*KI there were no tags that were present in all samples when tag length was 32 or 65 bp. At the lower prevalence thresholds *Ape*KI tended to have more tags that passed these thresholds than *Pst*I; however, this switched at higher prevalence. A greater proportion of reads were assigned when using *Pst*I than *Ape*KI at each filter level and tag length (Tables S1.1, S1.2). As the tag length decreased, the proportion of reads assigned increased, resulting in the greatest proportion of reads captured being 16 bp tags present in 10% or more of samples.

*Performance*

Overall there was little impact of tag size and filtering threshold on repeatability, correlation with methane yield and methane yield microbiability, particularly for *Pst*I (Tables S2.1 and S2.2). Tag lengths of 65bp were deemed the most appropriate due to the higher chance of getting a precise and accurate taxonomic assignment if that was desired, and a filter of 25% captured a larger proportion of reads than 50%. Results from both restriction enzymes showed there was a decrease in performance when there were too many (filter of 10%) or too few (filter of 100%) tags. The abundance of the tags was similar between *Ape*KI and *Pst*I, but the mean and maximum abundance were the same between the two restriction enzymes, however, *Pst*I haa a wider range than *Ape*KI, partially due to the greater number of tags for *Pst*I than *Ape*KI (Figure S2.1).

**Conclusions**

*Pst*I was less sensitive to changes in tag length and prevalence than *Ape*KI. Requiring a tag to be present in all samples was not appropriate for either enzyme at any tag length. Tags of length 65bp present in at least 25% of samples were selected for both *Ape*KI and *Pst*I in the main paper due to high repeatability estimate and moderately high relationships with methane yield.

**Table S2.1:** Comparison of different tag lengths and filtering criteria for *Ape*KI

| **Tag**  **Length**  **(Unique Tags)** | **Tag Prevalence (%)** | **Number**  **of Tags** | **Percent of Reads**  **Captured**  **(SD)** | **Principal Component** | **PC % Variance^1^** | **Cohort % Variance^2^** | **Repeatability^3^**  **(SE)** | **\|r_p_ (CH_4_ Yield)\|^4^** | **Microb.^5^**  **(SE)** |
| --- | --- | --- | --- | --- | --- | --- | --- | --- | --- |
| 65 bp  (86M) | 10 | 1,249,825 | 20.0 (3.5) | PC1 | 6.0 | 34.4 | 0.20 (0.09) | 0.35 (0.06) | 0.33 (0.08) |
|  |  |  |  | PC2 | 2.4 | 32.9 | 0.22 (0.09) | 0.46 (0.06) |  |
|  | 25 | 233,587 | 9.9 (2.5) | PC1 | 9.0 | 42.8 | 0.28 (0.09) | 0.36 (0.06) | 0.35 (0.09) |
|  |  |  |  | PC2 | 4.4 | 40.2 | 0.18 (0.09) | 0.48 (0.05) |  |
|  | 50 | 28,855 | 3.4 (1.4) | PC1 | 17.4 | 37.6 | 0.31 (0.08) | 0.43 (0.06) | 0.38 (0.09) |
|  |  |  |  | PC2 | 5.2 | 34.3 | 0.07 (0.09) | 0.15 (0.07) |  |
|  | 100 | 0 | 0 | PC1 | - | - | - | - | - |
|  |  |  |  | PC2 | - | - | - | - |  |
| 32 bp  (86M) | 10 | 2,756,402 | 50 (6.2) | PC1 | - | - | - | - | - |
|  |  |  |  | PC2 | - | - | - | - |  |
|  | 25 | 647,457 | 28.9 (5.3) | PC1 | 7.3 | 42.4 | 0.34 (0.08) | 0.39 (0.06) | 0.36 (0.09) |
|  |  |  |  | PC2 | 3.6 | 37.4 | 0.24 (0.09) | 0.49 (0.05) |  |
|  | 50 | 104,124 | 12.1 (3.3) | PC1 | 11.6 | 45.7 | 0.40 (0.08) | 0.47 (0.06) | 0.38 (0.08) |
|  |  |  |  | PC2 | 4.5 | 40.4 | 0.20 (0.09) | 0.36 (0.06) |  |
|  | 100 | 0 | 0 | PC1 | - | - | - | - | - |
|  |  |  |  | PC2 | - | - | - | - |  |
| 16 bp  (20M) | 10 | 3,692,140 | 79.4 (3.0) | PC1 | - | - | - | - | - |
|  |  |  |  | PC2 | - | - | - | - |  |
|  | 25 | 1,278,826 | 56.6 (4.0) | PC1 | 5.0 | 37.2 | 0.30 (0.09) | 0.41 (0.06) | 0.36 (0.09) |
|  |  |  |  | PC2 | 2.9 | 33.2 | 0.07 (0.09) | 0.38 (0.06) |  |
|  | 50 | 314,497 | 30.8 (3.5) | PC1 | 6.9 | 51.0 | 0.51 (0.07) | 0.51 (0.05) | 0.38 (0.08) |
|  |  |  |  | PC2 | 3.1 | 43.6 | 0.25 (0.09) | 0.50 (0.05) |  |
|  | 100 | 5 | 0.01 (0.04) | PC1 | 47.4 | 22.4 | 0.22 (0.09) | 0.35 (0.06) | 0.02 (0.01) |
|  |  |  |  | PC2 | 27.9 | 7.1 | 0.16 (0.09) | 0.03 (0.07) |  |

1. Percent of total metagenomic variance explained by PC1 or PC2
2. Percent of the variance in PC1 or PC2 explained by cohort
3. Percent of the variation in PC1 and PC2 (after adjusting for cohort) that is due to the permanent environmental effect
4. Absolute value of the correlation of PC1 and PC2 (after adjusting for cohort) with methane yield
5. Microbiability: Proportion of the variance in methane yield that can be attributed to the microbial relationship matrix

-: Not run because too many tags for a high-throughput approach (>2M) or too few (0) tags

**Table S2.2:** Comparison of different tag lengths and filtering criteria for *Pst*I

| **Tag**  **Length**  **(Unique Tags)** | **Tag Prevalence (%)** | **Number**  **of Tags** | **Percent of Reads**  **Captured**  **(SD)** | **Principal Component** | **PC % Variance^1^** | **Cohort % Variance^2^** | **Repeatability^3^**  **(SE)** | **\|r_p_ (CH_4_ Yield)\|^4^** | **Microb.^5^**  **(SE)** |
| --- | --- | --- | --- | --- | --- | --- | --- | --- | --- |
| 65 bp  (86M) | 10 | 1,295,546 | 66.6 (5.7) | PC1 | 6.3 | 9.4 | 0.24 (0.09) | 0.19 (0.04) | 0.40 (0.08) |
|  |  |  |  | PC2 | 4.0 | 85.2 | 0.32 (0.09) | 0.13 (0.07) |  |
|  | 25 | 502,900 | 53.3 (5.9) | PC1 | 6.3 | 54.8 | 0.49 (0.07) | 0.43 (0.06) | 0.41 (0.08) |
|  |  |  |  | PC2 | 3.8 | 52.9 | 0.50 (0.07) | 0.46 (0.06) |  |
|  | 50 | 163,078 | 36.8 (5.5) | PC1 | 7.8 | 60.7 | 0.54 (0.07) | 0.53 (0.05) | 0.40 (0.08) |
|  |  |  |  | PC2 | 4.0 | 59.7 | 0.71 (0.05) | 0.63 (0.05) |  |
|  | 100 | 3 | 0.01 (0.01) | PC1 | 61.1 | 11.2 | 0.34 (0.08) | 0.26 (0.07) | 0.01 (0.01) |
|  |  |  |  | PC2 | 31.1 | 27.9 | 0.17 (0.09) | 0.06 (0.07) |  |
| 32 bp  (86M) | 10 | 1,386,448 | 85.3 (3.7) | PC1 | 6.9 | 7.3 | 0.21 (0.09) | 0.14 (0.04) | 0.41 (0.08) |
|  |  |  |  | PC2 | 4.3 | 82.5 | 0.44 (0.08) | 0.34 (0.06) |  |
|  | 25 | 605,628 | 72.3 (5.4) | PC1 | 6.3 | 54.1 | 0.50 (0.07) | 0.44 (0.05) | 0.41 (0.08) |
|  |  |  |  | PC2 | 3.9 | 56.2 | 0.31 (0.09) | 0.30 (0.06) |  |
|  | 50 | 221,991 | 53.6 (6.1) | PC1 | 7.6 | 61.1 | 0.56 (0.07) | 0.55 (0.05) | 0.40 (0.08) |
|  |  |  |  | PC2 | 4.8 | 78.9 | 0.00 (0.00) | 0.07 (0.07) |  |
|  | 100 | 7 | 0.06 (0.04) | PC1 | 48.0 | 32.8 | 0.34 (0.08) | 0.36 (0.06) | 0.05 (0.04) |
|  |  |  |  | PC2 | 18.5 | 28.2 | 0.21 (0.09) | 0.21 (0.07) |  |
| 16 bp  (20M) | 10 | 1,014,087 | 96.0 (1.3) | PC1 | 7.5 | 7.1 | 0.17 (0.09) | 0.12 (0.04) | 0.41 (0.08) |
|  |  |  |  | PC2 | 4.1 | 76.9 | 0.58 (0.06) | 0.51 (0.05) |  |
|  | 25 | 571,088 | 89.0 (2.7) | PC1 | 6.0 | 52.7 | 0.53 (0.07) | 0.48 (0.05) | 0.41 (0.08) |
|  |  |  |  | PC2 | 3.6 | 57.7 | 0.22 (0.09) | 0.21 (0.07) |  |
|  | 50 | 275,411 | 75.2 (4.1) | PC1 | 7.3 | 61.3 | 0.60 (0.06) | 0.58 (0.05) | 0.41 (0.08) |
|  |  |  |  | PC2 | 4.1 | 79.9 | 0.00 (0.00) | 0.11 (0.07) |  |
|  | 100 | 40 | 0.5 (0.3) | PC1 | 32.4 | 36.9 | 0.36 (0.08) | 0.38 (0.06) | 0.27 (0.08) |
|  |  |  |  | PC2 | 11.1 | 21.0 | 0.18 (0.09) | 0.12 (0.07) |  |

1. Percent of total metagenomic variance explained by PC1 or PC2
2. Percent of the variance in PC1 or PC2 explained by cohort
3. Percent of the variation in PC1 and PC2 (after adjusting for fixed effects) that is due to the permanent environmental effect
4. Absolute value of the correlation of PC1 and PC2 (after adjusting for fixed effects) with methane yield
5. Microbiability: Proportion of the variance in methane yield that can be attributed to the microbial relationship matrix


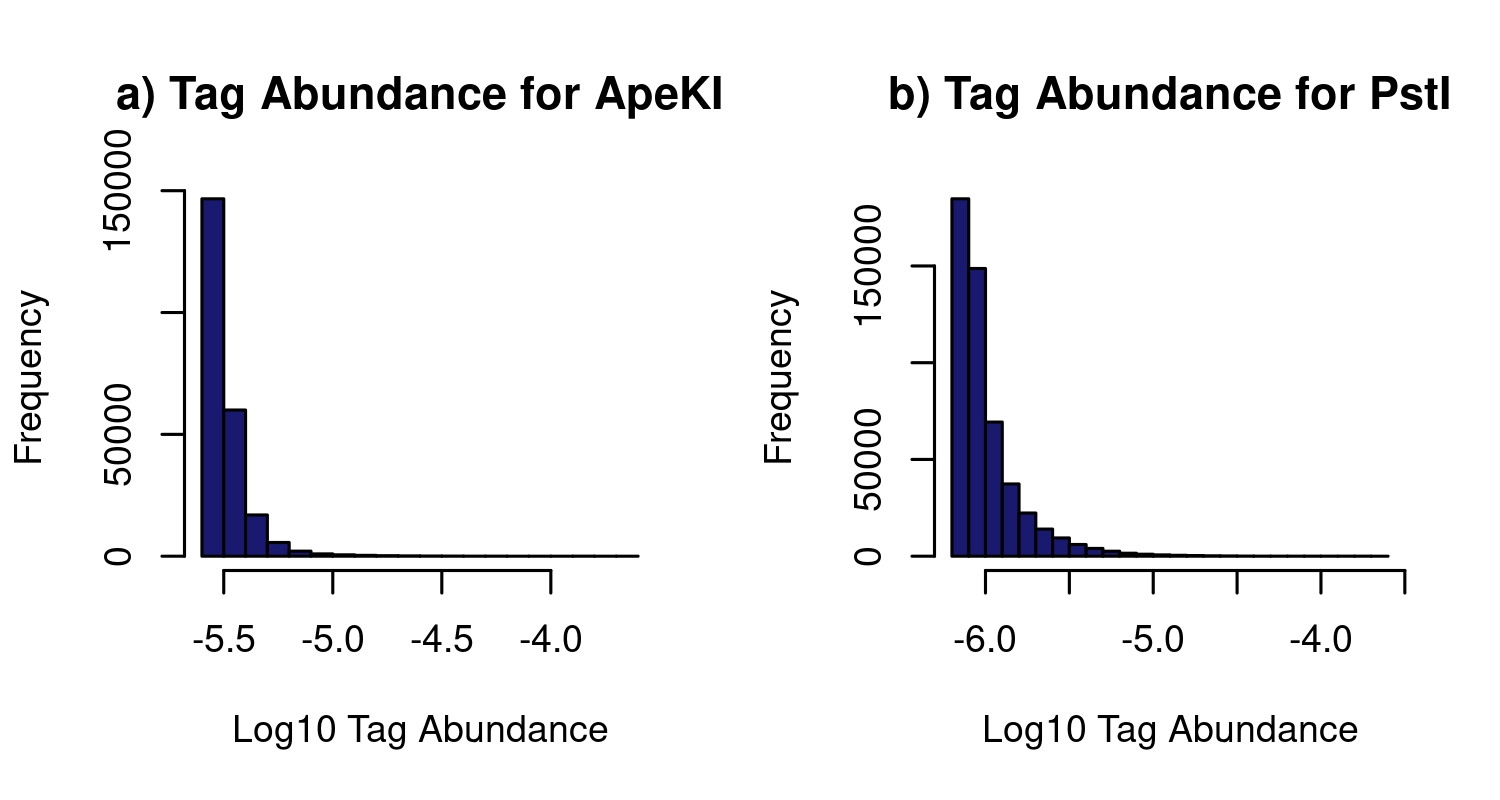


**Figure S2.1: Tag abundances for *Ape*KI (a) and *Pst*I (b) for 65 bp tags that are present in at least 25% of samples.** Average tag abundances from the 233,587 tags generated using *Ape*KI (a) and the 502,900 tags generated using *Pst*I. *Ape*KI captured. Tag abundances were calculated as the proportion of total reads that were assigned to a tag: 9.9% of reads for *Ape*KI and 53.3% for *Pst*I.
